# Prevalence, Resistance Patterns and Biofilm Production Ability of Bacterial Uropathogens from Cases of Community-Acquired Urinary Tract Infections in South Italy

**DOI:** 10.3390/pathogens12040537

**Published:** 2023-03-29

**Authors:** Angela Maione, Emilia Galdiero, Luigi Cirillo, Edvige Gambino, Maria Assunta Gallo, Francesca Paola Sasso, Arianna Petrillo, Marco Guida, Marilena Galdiero

**Affiliations:** 1 Department of Biology, University of Naples ’Federico II’, Via Cinthia, 80126 Naples, Italy; 2 Department of Neurosciences, Reproductive Sciences and Odontostomatology, University of Naples “Federico II”, 80131 Naples, Italy; 3 Ciro Diagnostic Center, 80055 Naples, Italy; 4 Department of Dermatology and Venereology, University of Rome “La Sapienza”, 00161 Rome, Italy; 5 Università degli Studi di Milano, 20122 Milan, Italy; 6 Department of Experimental Medicine, University of Campania “Luigi Vanvitelli”, 81100 Naples, Italy

**Keywords:** urinary tract infection, uropathogens, antibiotics, antimicrobial resistance, biofilm, empirical therapy

## Abstract

Community-acquired urinary tract infections represent the most common infectious diseases in the community setting. Knowing the antibiotic resistance patterns of uropathogens is crucial for establishing empirical treatment. The aim of the current study is to determine the incidence of the causative agents of UTIs and their resistance profiles. Patients of all ages and both sexes were enrolled in the study, and admitted to San Ciro Diagnostic Center in Naples between January 2019 and Jun 2020. Bacterial identification and antibiotic susceptibility testing were carried out using Vitek 2 system. Among the 2741 urine samples, 1702 (62.1%) and 1309 (37.9%) were negative and positive for bacterial growth, respectively. Of 1309 patients with infection, 760 (73.1%) were females and 279 (26.9%) were males. The greatest number of positive cases were found in the in the elderly (>61 years). Regarding uropathogens, 1000 (96.2%) were Gram-negative while 39 (3.8%) were Gram-positive strains. The three most isolated pathogenic strains were *Escherichia coli* (72.2%), *Klebsiella pneumoniae* (12.4%), and *Proteus mirabilis* (9.0%). Strong biofilm formation ability was observed in about 30% of the tested isolates. The low resistance rates recorded against nitrofurantoin, fosfomycin, piperacillin–tazobactam, and gentamicin could suggest them as the most appropriate therapies for CA-UTIs.

## 1. Introduction

Urinary tract infections (UTIs) are a severe public health issue and are usually due to a range of pathogens, including Gram-negative and Gram-positive bacteria, as well as certain yeasts. UTIs are common microbial diseases that involve any part of the urinary system, including the kidneys, renal pelvis, ureters, bladder, and urethra [1]. UTIs are spread globally, leading to significant morbidity and direct and indirect social and economic impacts [2]. According to global surveillance studies, UTIs represent the second most frequent infection type, after respiratory tract infections [3].

Worldwide, UTIs affect about 150 million people each year [4]. The healthcare costs exceed USD 6 billion with over 1 million medical tests and 100,000 hospitalizations every year [5]. Increased antibiotic resistance and recurrence rates of uropathogens (UPs) pose a substantial economic burden worldwide [6,7].

UTIs can be classified into hospital- (HA-UTIs) and community-acquired urinary tract infections (CA-UTIs). HA-UTIs occur 48 h after hospitalizations or 3 days after resignation, while CA-UTIs appear in a community setting or after less than 48 h of hospital admission and represent one of the most frequently encountered bacterial infections in everyday health care [8,9]. The incidence of UTIs varies according to several factors: (i) age; (ii) gender; (iii) use of catheters; (iv) admission to hospital; (v) comorbidities; and (vi) prolonged use of antibiotics [10]. A UTI occurs when the pathogen enters the urinary tract system and presents in the urine in quantities of more than 105 colony-forming units per milliliter (CFU/mL) [11,12]. According to various studies, UTIs are mostly caused by both Gram-negative (approximately 80–90%) and Gram-positive bacteria (10–20%) [11]. Several studies identified Enterobacterales such as *Escherichia coli*, *Klebsiella pneumoniae*, *Proteus mirabilis*, Citrobacter, and Enterobacter as common etiological agents of CA-UTIs. Other less common causes include *Pseudomonas aeruginosa*, *Acinetobacter baumannii*, *Staphylococcus aureus*, *Enterococcus faecalis*, and the yeast *Candida albicans* [13,14]. UTIs can be asymptomatic or symptomatic and are diagnosed through the analysis of the patient’s symptoms and microbiological tests [15]. The most common clinical signs include (i) dysuria; (ii) hesitation; (iii) frequency; (iv) hematuria; (v) urgency; and (vi) back and abdominal pain [16]. Cultural examination, bacterial identification, and antibiograms represent the gold standard to diagnose UTIs [17]. The report of the causative agent leading to UTI and the related resistance pattern are provided approximately 48 h after sampling [18]. Therefore, the treatment of CA-UTIs is mainly empirical, based on the limited data regarding the spectrum of etiological agents and their antibiotic resistance profiles [19]. In addition, in most cases, culture and antibiograms cost more than antibiotic therapy [19]. These factors complicate the empirical treatment of the infection, due to mismanagement of the antibiotic prescription with a consequent emergence of antimicrobial resistance (AMR) [20,21]. Multidrug resistance (MDR) is one of the greatest problems for public health. In the last decades, due to the spread of MDR, UPs have become a serious clinical problem, especially in developing countries, as they lead to excessive use of broad-spectrum antibiotics, prolonged hospitalization, and consequent high costs of treatment [22]. Indeed, in Europe, the treatment of UTIs covers 9% of antibiotic prescriptions [23]. Failure of antibiotic treatment and negative therapeutic outcomes may lead to the development of serious clinical complications [24]. The high prevalence of CA-UTIs and the growing problem of antibiotic resistance underline the need for continuous local surveillance of the antibiotic resistance profiles of UPs. An adequate knowledge of local antibiotic resistance trends is essential to improve empirical antibiotic treatment of UTIs [25,26]. The relationship between AMR and biofilms production is inconsistently reported across the literature. This suggest that the true correlation between these two aspects is still unknown. Biofilms are dynamic communities of commensal and pathogenic microorganisms with transitions from a planktonic state to a sessile state embedded in an extracellular matrix. Approximately 60–80% of microorganisms causing UTIs have been reported to be capable of forming both single- and multispecies biofilms [25]. In medicine, the important role of biofilm-producer microorganisms is well recognized. In fact, they play an essential role in the antibiotic resistance process as they protect microorganisms against antimicrobial penetration, consequently altering the microenvironment [27].

As the resistance to antimicrobials in microorganisms isolated from patients with UTIs can differ from region to region and patient to patient, a deeper understanding of the community acquired microorganisms in our population is the first point to focus on. The purpose of this study was to investigate the distribution, the related antibiotic resistance profiles, and biofilm formation ability of bacteria isolated from urinary tract infections (UTIs) to improve the efficacy of the empirical treatment of these infections. To the best of our knowledge, this study is the first to shed some light on the virulence factors, such as the ability to form biofilms of community-associated multidrug-resistant UPs in south Italy.

## 2. Materials and Methods

### 2.1. Samples Collection

This study was conducted on a total of 2741 urine samples collected from patients of the San Ciro Diagnostic Center in Naples in the period between January 2019 and June 2020. Each patient provided midstream specimens of urine (MSU) to the bacteriology laboratory for processing. The samples were collected in sterile urine collecting bottles, transported to the laboratory on ice in darkness, and microbiological assays were initiated on the day of sampling.

### 2.2. Inclusion and Exclusion Criteria

The selection of the subjects in this study was in accordance with CDC guidelines. The recruited symptomatic or asymptomatic patients met the following inclusion criteria: (i) one or more symptoms of UTIs as well as severe urinary excretion with frequent urination, dysuria, incomplete emptying of the bladder, suprapubic pain, occasional presence of leukocytes or blood in the urine and, finally, a positive culture with ≥10^5^ CFU/mL colonies by a monomorphic growth; (ii) patients who had received antibiotic treatment more than two weeks prior to the test.

Exclusion criteria were (i) female patients in their menstrual period; (ii) patients with antibiotic administration in the last two weeks; (iii) cultures with CFU/mL less than 104 CFU/mL; and (iv) catheterized patients.

Information on patients including name, age, sex, clinical history, and treatment history were collected with the patient’s consent.

### 2.3. Bacterial Culture, Bacterial Identification, and Antibiotic Susceptibility Test

Specimens were plated on Blood agar, MacConkey medium, CNA blood agar, and Sabouraud Glucose agar (OXOID) at 37 °C for 18–36 h unless otherwise specified [22]. Bacteriuria was defined when the bacterial load was greater than 10^5^ CFU/mL. Species identification and antibiotic sensitivity tests were performed through Vitek 2 (BioMe’rieux, France) with identification cards (ID-GN for Gram-Negative, ID-GP for Gram-positive, YST for yeast) and the AST-659 (for staphylococci), AST-658 (for enterococci), AST-STO3 (for *S. agalactiae*), and AST-397 (for Gram-Negative), following the manufacturer’s recommendations. Strains were stored in a Tryptose Soya Broth (TSB) with 20% glycerol at −70 °C.

The tested antibiotics in this study were ampicillin, norfloxacin, amoxicillin–clavulanic acid, ciprofloxacin, trimethoprim–sulfamethoxazole, cefotaxime, ceftazidime, gentamicin, cefepime, piperacillin–tazobactam, fosfomycin, nitrofurantoin, ertapenem, imipenem, amikacin, and meropenem. Results as “susceptible” or “resistant” were interpreted according to EUCAST guidelines [28].

Multidrug resistance (MDR) was defined as resistance against at least four antibiotics, while non-MDR was defined as resistance against three or more distinct classes of antibiotics.

### 2.4. Assay of Biofilm Formation

The ability of isolates to form biofilms was measured using the 96-well microtiter plate method as described previously [29,30], using TSB (OXOID, Basingstoke, UK) as growth media and detected using the crystal violet staining method for total biofilm biomass determination, according to Stepanović et al. [31]. A total of 100 mL of each culture at a concentration of 1 × 106 CFU mL^−1^ was pipetted into wells of a polystyrene 96-well microplate. After 24 h at 37 °C, microplates were rinsed three times with PBS, fixed with 99% methanol for 15 min, and air-dried before adding 200 μL of 1% crystal violet (CV) (Sigma-Aldrich, St. Louis, MO, USA) to each well. After incubation for 15 min at room temperature, wells were washed three times with PBS, and 300 μL of acetic acid (30% *v*/*v*) was added to each well. Plates were read at 570 nm using a plate reader (SYNERGY H4 BioTek, BioTek Instruments, Agilent Technologies, Winooski, VT 05404, USA). To quantify the biofilm formation abilities, the optical density cut off value (ODc) was calculated as the three standard deviations above the mean OD of the negative control. The final OD value of the isolate was interpreted as negative (OD ≤ ODc), weak (ODc ≤ OD ≤ 2ODc), moderate (2ODc < OD ≤ 4ODc), or strong (4ODc < OD) biofilm former [31].

## 3. Results

### 3.1. UTI Prevalence in Studied Patients

During the study period, 1 year and a half, 2741 urine samples were processed. The infectious condition was established based on the patient’s clinical signs, and the presence of leukocytes and bacteria in the urine. In total, 1039 (37.9%) of these were positive for bacterial growth and each positive sample was represented by one bacterial isolate, whereas 1702 (62.1%) were negative as shown in Table 1. In relation to the gender of positive patients, as summarized in Table 1, a higher prevalence of female infections was detected for all the age groups, 760 (73.1%) females and 279 (26.9%) males. Regarding the distribution of infection among patient age groups, a higher prevalence of UTIs was recorded in the elderly, both in men and women, with a frequency of 47.7% (>61 years), followed by late adulthood (46–60 years) 19.7%, and young adults (19–45 years) 13.7%. The least affected group was early childhood (2–5 years), showing the lowest frequency of UTIs, 2.7%. Adolescents (13–18 years) were responsible for 5.9% of the infections, late childhood (6–12 years) for 3.3%, and infants (<1 years) for 7% (Table 1). The most representative microorganisms isolated from the 1039 community samples were 39 (3.8%) Gram-positive and 1000 (96.2%) Gram-negative (Table 1).

### 3.2. Bacteria Implicated in UTI

Table 2 summarizes the distribution of bacterial species in positive urine samples. For Gram-positive bacteria, *Enterococcus faecalis* (*E. faecalis*) was the most isolated bacterium (1.3%), succeeded by *Enterococcus gallinarum* (*E. gallinarum*) (0.5%), *Staphylococcus aureus* (*S. aureus*) (0.5%), *Enterococcus faecium* (*E. faecium*) (0.5%), *Streptococcus pyogenes* (*S. pyogenes*) (0.3%), *Staphylococcus xylosus* (*S. xylosus*) (0.2%), *Streptococcus alactolyticus* (*S. alactolyticus*) (0.1%), *Streptococcus agalactiae* (*S. agalactiae*) (0.1%), *Staphylococcus saprophyticus* (*S. saprophyticus*) (0.1%), *Staphylococcus haemolyticus* (*S. haemolyticus*) (0.1%), and *Enterococcus durans* (*E. durans*) (0.1%) (Table 2). For Gram-negative bacteria, *Escherichia coli* (*E. coli*) was the most frequently isolated bacterium (72.2%), followed by *Klebsiella pneumoniae* (*K. pneumoniae*) (12.4%), *Proteus mirabils* (*P. mirabilis*) (9.0%), *Pseudomonas aeruginosa* (*P. aeruginosa*) (1.2%), *Citrobacter koseri* (*C. koseri*) (0.7%), *Serratia marcescens* (*S. marcescens*) (0.2%), *Klebsiella oxytoca* (*K. oxytoca*) (0.2%), *Raoultella planticola* (*R. planticola*) (0.1%), *Raoultella ornithinolytica* (*R. ornithinolytica*) (0.1%), *Enterobacter aerogenes* (*E. aerogenes*) (0.1%), *Acinetobacter baumannii* (*A. baumannii*) (0.1%), and *Lelliottia amnigena* (*L. amnigena*) (0.1%) (Table 2).

### 3.3. Prevalence of Antibiotic Resistance among Identified Uropathogens

*E. coli*, *K. pneumoniae*, and *P. mirabilis* were the most encountered bacterial strains and their antibiotic resistance profiles were analyzed (Table 3). These isolated bacteria exhibited a high rate of resistance to the antibiotics tested. Data showed that *E. coli* exhibited a resistance rate greater than 40.7% to three antibiotics: ampicillin, norfloxacin, and amoxicillin–clavulanic acid. In contrast, susceptibility levels above 92.8% were recorded for piperacillin–tazobactam, fosfomycin, nitrofurantoin, ertapenem, imipenem, amikacin, and meropenem. A total of 21.6% of *E. coli* strains exhibited an extended spectrum β-Lactamase (ESBL) phenotype. As with *E. coli*, *K. pneumoniae* showed 100%, 40.6%, and 42.2% resistance to ampicillin, norfloxacin, and amoxicillin–clavulanic acid, respectively. Susceptibility rates greater than 93% were detected for gentamicin, ertapenem, imipenem, amikacin, and meropenem. Among these strains, 21.7% were ESBL producing *K. pneumoniae*. Regarding *P. mirabilis*, the highest levels of resistance were found for ampicillin (66.7%) and trimethoprim–sulfamethoxazole (40.9%). The latter showed sensitivity rates greater than 92.5% towards ertapenem, amikacin, and meropenem. The uropathogens, leading to CA-UTIs, showed resistance levels greater than 40.6% for ampicillin, norfloxacin, and amoxicillin/clavulanic acid.

### 3.4. Biofilm Formation of UPs Isolates

The ability to form biofilm represents one of the major virulence factors. Therefore, 60 UPs isolates selected on resistance to certain classes of antibiotics were examined using microtiter plates with crystal violet staining. The reference strains *E. coli* ATCC 25922, *K. pneumoniae* ATCC 13883, and *P. mirabilis* ATCC 35659/7002 were used as positive controls. As shown in Table S1, of twenty *E. coli* isolates, six (30%), eight (40%), and three (15%) isolates were strong biofilm producers, moderate biofilm producers, and weak biofilm producers, respectively. Contrarily, three (15%) isolates were not biofilm producers. The rate of biofilm production in multidrug-resistant (MDR) *E. coli* isolates was 80%. Of the twenty *K. pneumoniae* isolates (Table S1), five (25%), seven (35%), and seven (35%) isolates resulted as strong, moderate, and weak biofilm producers, respectively, while one (5%) isolate was not a biofilm producer. The rate of *K. pneumoniae* MDR isolates was 50%.

Of *P. mirabilis* isolates (Table S1), five (25%), five (25%), and five (25%) isolates resulted as strong, moderate, and weak biofilm producers, respectively, and 25% (five) did not produce biofilm. The rate of *Proteus mirabilis* MDR was 45%.

Table 4 shows the correlation between antibiotic resistance and biofilm production in MDR and non-MDR isolates. The distribution of isolates with various biofilm-forming capacities demonstrated significant differences between MDR and non-MDR groups, except for isolates that formed moderate biofilm. Based on OD (λ = 570 nm) measurements of 60 isolates examined, 55% (n = 33) were MDR, and all of them (100%) were able to form biofilm, while the 43.3% (n = 26) were non-MDR, and 62.5% of them were able to form biofilm. Among MDR isolates, 45% of them were strong biofilm producers and among non-MDR isolates, only 5.8% produced a strong biofilm. Moreover, 42.4% and 12.1% of MDR isolates showed moderate and weak biofilm abilities, respectively. The results showed instead that 25.9% and 33.3% of non-MDR isolates were moderate and weak biofilm producers, respectively.

## 4. Discussion

Antimicrobial drug resistance is a global problem with severe public health implications. Urinary tract infections (UTIs) are commonly found infections in all age groups, with relevant clinical impact due to their high morbidity and mortality [4]. UTIs have the highest rank among all diseases in terms of the number of antibiotic prescriptions used to treat infected individuals, as they are usually treated empirically [32]. Over the time, the high rates of antimicrobial resistance reported among uropathogens have progressively increased worldwide, making UTIs one of the most encountered infectious diseases in medical practice, causing relevant morbidity [2]. The knowledge of microorganisms and their antimicrobials together with the study of resistance patterns in the local environment are essential to provide more personalized and effective treatments [33,34].

Several evidences demonstrated local variations in UTIs etiology and related antibiotic resistance profiles [18,35,36]. Furthermore, due to the variety of causative agents and antibiotic resistance patterns between regions, empiric therapy for UTIs is, presently, based on local susceptibility data. Hence, for the successful empirical treatment of UTIs, a knowledge of uropathogens and their resistance to antibiotics is essential [37]. The present study shows the causative agents of CA-UTIs and their antibiotic resistance profiles in the community setting (south Italy, Naples), providing important data that could be useful in the drawing of local guidelines for the treatment of CA-UTIs.

Out of 2741 urine samples, 37.9% of patients exhibited a significant bacteriuria. Our data were comparable to a study conducted in Serbia by Donkor et al., who reported a prevalence rate of 34.4% [19]. In contrast, a lower prevalence of infections was found in Accra (10.1%). The prevalence of CA-UTIs was higher in females (73.1%) than in males (26.9%), in accordance with several studies [38,39]. The higher occurrence in female patients is justified by the anatomy of the reproductive system (short urethra) [40,41]. The greatest number of positives was found in the elderly (>61), followed by patients aged 46 to 60 years. The predisposing factors are (i) functional disorders of the prostate and hormonal changes in females; (ii) incontinence; (iii) structural alterations of the urinary tract; (iv) comorbidities (diabetes, dialysis, etc.) [10].

Gram-negative bacteria were the main cause of CA-UTIs (96.2%), in particular, *E. coli* (72.2%) was the predominant pathogen, followed by *K. pneumoniae* (12.4%) and *P. mirabilis* (9.0%). The high prevalence of *E. coli* was documented in reports from several states [42]. In particular, Donkor et al. found *E. coli* (48.4%) and *K. pneumoniae* (16.1%) as the main causative agents of CA-UTIs, while *P. mirabilis* contributed only in part in Accra (Ghana) [19]. Moreover, Odoki et al. documented the incidence of 41.9, 31.4, and 11.6% for *E. coli, Staphylococcus aureus*, and *K. pneumoniae*, respectively, whereas *P. mirabilis* was responsible for only 3.5% of CA-UTI cases in the Bushenyi District (Uganda) [43].

International guidelines for the treatment of UTIs recommend nitrofurantoin, trimethoprim–sulfamethoxazole, fosfomycin, fluoroquinolones, and beta-lactams [41,44].

Resistance rates of trimethoprim–sulfamethoxazole, ciprofloxacin, and amoxicillin–clavulanic acid are worrying. Antibiotic resistance rates greater than 30.8% were associated with these antibiotics. In particular, trimethoprim–sulfamethoxazole resistance levels of 30.8, 34.1, and 41.3% were recorded for *E. coli*, *K. pneumoniae*, and *P. mirabilis*, respectively. Erdem et al. reported higher trimethoprim–sulfamethoxazole resistance rates for *E. coli* (43.4%) and *K. pneumoniae* (56.3%) in Tekirdag (Turkey) [45]. For *P. mirabilis*, de Oliveira et al. documented that 78.1% were susceptible to this antibiotic in Londrina (Brazil) [46]. Regarding ciprofloxacin resistance rates, 35.2, 32.6, and 39.1% were associated with *E. coli*, *K. pneumoniae*, and *P. mirabilis*. In Tekirdag (Turkey), resistance levels of ciprofloxacin were highest for *E. coli* (56.7%) and *K. pneumoniae* (60.0%) (30166811). Moreover, low susceptibility rates to ciprofloxacin were detected in 96.7% of the *P. mirabilis* strains isolated in Londrina (Brazil) [46]. Our results are in line with our regional reports [47] and recent data from other Italian regions. In fact, higher trimethoprim–sulfamethoxazole and ciprofloxacin sensitivity levels were reported in Milan (Italy). Paris et al. documented trimethoprim–sulfamethoxazole sensitivity levels of 76.0, 83.8, and 61% for *E. coli*, *K. pneumoniae*, and *P. mirabilis*, respectively. For ciprofloxacin, 75, 80, and 59.8% of *E. coli*, *K. pneumoniae*, and *P. mirabilis* were sensitive to ciprofloxacin, respectively [44]. Regarding amoxicillin–clavulanic acid, susceptibility levels were lower in the Milan area. In particular, Paris et al. reported susceptibility rates greater than 75.8% for *E. coli*, *K. pneumoniae*, and *P. mirabilis* [44]. Low susceptibility rates (>70%) to nitrofurantoin, fosfomycin, piperacillin–tazobactam, and gentamicin suggested their efficient use in the treatment of these infections, in accordance with the studies cited above [33,34]. The interesting levels of sensitivity (S ≤ 11%) towards cefepime, nitrofurantoin, and fosfomycin suggest these antibiotics may be best for empirical treatment towards the strains most commonly implicated in CA-UTIs [48].

The ability to form biofilms is important in the pathogenesis of microbial infections as it implies their ability to resist antimicrobial agents and become persistent sources of chronic infections. In fact, biofilm formation has profound implications for public health because it is a survival mechanism that helps microorganisms adapt to adverse environmental conditions. Bacteria within the biofilm behave differently from their planktonic counterparts, especially regarding antibiotic susceptibility, which causes limitations in conventional antibiotic therapies.

The biofilm-forming ability of UPs enhances their ability to persist in the urinary tract environment, evading the immune system, and it has been suggested to certainly play a role in recurrent infections and in the increasing emergence of MDR. Previous studies have also highlighted the role of biofilm formation in UTIs [49].

The observations in our isolates are similar to those reported previously [50,51,52,53], suggesting that the acquisition of multiple resistance in UPs isolates is strongly associated with their biofilm formation capacities. Abdagire et al. showed that 43.4% of the uropathogens were in vitro positive for biofilm formation [49], confirming that the result of the potentiality to anticipate the formation of the biofilm among the population studied allows the choice of the most suitable and effective antibiotic therapy, with health advantages to the patients and financial benefits to the institutions.

*E. coli* are the leading cause of urinary tract infections, causing over 95% of community-acquired infections. They are able to survive by establishing urinary tract infections involving a wide variety of virulence factors including the ability to form biofilms, especially in MDR isolates as reported in our research. In fact, we reported that about 60% of analyzed isolates formed strong/moderate biofilms. Hence, effective strategies for the management of biofilm forming *E. coli* are crucial, as ineffective strategies may lead to relapses in untreatable UTIs.

Overall, 5–10% of UTIs are caused by *K. pneumoniae*, which is one of the high-priority species due to a growing global problem of antimicrobial resistance [54]. Our results also showed that the *K. pneumoniae* ability to form strong/moderate biofilms was about 65%.

*P. mirabilis* showed the potential to create biofilm in various environments, including on biological surfaces causing urine obstruction in the bladder, recurrent bacteriuria, fever, sepsis, and shock. Between the isolates analyzed, about 50% were able to form biofilms that could surely contribute to disease [55].

The difference in resistance patterns of the bacterial isolates is probably connected to geographical region, the time of study, and clinical practice [56]. According to Sanchez et al., and Alves et al., biofilm formation increases the resistance profile of the organisms, and the strains that are capable of forming biofilm are mostly MDR phenotypes [57]. Biofilm formation by clinical isolates and the implications in chronic infections [49] show that a higher prevalence of MDR in biofilm producing strains could be due to the transfer of resistant genes [58]. A strong connection between biofilm formation and the development of resistance to particular drugs, such as ampicillin, ciprofloxacin, and norfloxacin, has been suggested by Tewawong et al. 2020 [59] for UPs isolates showing a possible relationship between their biofilm-forming ability and resistance profiles.

In this study, biofilm formation capability among the three different species of UPs was another important area of concern, contributing to the potential pathogenesis, drug resistance, and infection recurrence. Here, it was underlined that there was a strong connection not only between the biofilm formation ability and the development of multidrug resistance, but also regarding MDR UPs having a major capacity to be strong biofilm producers. According to our results, even if it is difficult to formulate definitive conclusions on this topic due to conflicting reports regarding the association between biofilm formation and the development of MDR in UPs, we could corroborate that there is a possible association of UPs with multi-antibiotic resistance and high rates of biofilm production in our country.

Although some limitations have to be considered, such as the fact that our study is limited to a single clinical service, or the lack of accurate information from the patients involved in the study, our data are important in order to gain insight into the widespread uropathogens in south Italy, as UTIs are one of the most frequently encountered bacterial infections in everyday healthcare.

## 5. Conclusions

In conclusion, our data show that the surveillance of the local etiology of UTIs and antibiotic susceptibility are essential to guide clinicians in establishing empirical therapy, as the prevalence of pathogens and their resistance to antibiotics vary over time and between geographical regions. This study promotes information on the current state in our country, in order to implement and establish new guidelines for the correct use of antibiotics.

## Figures and Tables

**Table 1:** Demographics data and characteristics of studied patients in UTI. Results were expressed as number and percentage.

Character		n (%)	
No pathogenic bacteria		1702 (62.1)	
Pathogenic bacteria		1039 (37.9)	
Gram +		39 (3.8)	
Gram −		1000 (96.2)	
Gender		n (%)	
Female		760 (73.1)	
Male		279 (26.9)	
Age Groups n (%)			
Male	Female	Tot.	Age
37 (13.3)	35 (4.6)	73 (7.0)	<1
10 (7.3)	18 (2.4)	28 (2.7)	2–5
12 (4.3)	22 (2.9)	34 (3.3)	6–12
19 (6.8)	42 (5.5)	61 (5.9)	13–18
46 (16.1)	97 (12.8)	142 (13.3)	19–45
23 (8.2)	182 (24.0)	205 (19.7)	46–60
184 (66.0)	312 (41.0)	4 96 (47.7)	>61

**Table 2:** Distribution of microorganisms in positive samples of urine.

Species	Gram Classification	Number	Prevalence (%)
*E. coli*	negative	750	72.2
*K. pneumoniae*	negative	128	12.3
*P. mirabilis*	negative	93	8.9
*E. fecalis*	negative	14	1.3
*P. aeruginosa*	negative	12	1.2
*C. koseri*	negative	8	0.7
*R. plancticola*	negative	1	0.1
*E. durans*	positive	1	0.1
*L. amnigena*	positive	1	0.1
*S. alactolycus*	positive	1	0.1
*S. agalactiae*	positive	1	0.1
*S. aureus*	positive	5	0.5
*E. gallinarum*	positive	5	0.5
*E. faecium*	positive	5	0.5
*S. pyogenes*	positive	3	0.3
*E. aerogenes*	negative	1	0.1
*S. marcescens*	negative	2	0.2
*K. oxytoca*	negative	2	0.2
*S. xylosus*	positive	2	0.2
*R. ornithiolytica*	negative	1	0.1
*S. saprophyticus*	positive	1	0.1
*A. baumannii*	negative	1	0.1
*S. haemolyticus*	positive	1	0.1

**Table 3:** Antibiotic resistance rates of *E. coli*, *K. pneumoniae*, and *P. mirabilis* isolates.

Bacteria	Antimicrobial Agents Tested n (%)															
	Amp	Nor	Amc	Cip	Sxt	Ctx	Caz	Gm	Fep	P/T	Fos	Nit	Ert	Imi	Ak	Mem
*E. coli*	485	358	305	264	231	173	143	104	79	55	49	23	6	1	1	1
	(64.7)	(47.7)	(40.7)	(35.2)	(30.8)	(23.2)	(19.1)	(13.9)	(10.5)	(7.3)	(6.5)	(3.1)	(0.8)	(0.1)	(0.1)	(0.1)
*K. pneumoniae*	128	52	54	42	44	31	22	9	15	20	31	-	3	2	2	2
	(100)	(40.6)	(42.2)	(32.8)	(34.4)	(24.2)	(17.1)	(7)	(11.7)	(15.6)	(24.2)		(2.3)	(1.6)	(1.6)	(1.6)
*P. mirabilis*	62	-	35	36	38	21	16	24	9	8	27	24	7	8	4	2
	(66.7)		(37.6)	(38.7)	(40.9)	(22.6)	(17.2)	(26.1)	(9.7)	(8.6)	(29)	(25.8)	(7.5)	(8.6)	(4.3)	(2.2)
Total (971)	675	410	394	342	313	225	195	137	103	83	107	46	16	11	7	5
	(69.5)	(46.7)	(40.6)	(35.2)	(32.2)	(23.2)	(20.1)	(14.1)	(10.6)	(8.5)	(11.0)	(4.7)	(1.7)	(1.1)	(0.7)	(0.5)

Abbreviation: Amp, ampicillin; Nor, norfloxacin; Amc, amoxicillin/clavulanic acid; Cip, ciprofloxacin; Sxt, trimethoprim/sulfamethoxazole; Ctx, cefotaxime; Caz, ceftazidime; Gm, Gentamycin; Fep, Cefepime; P/T, Piperacillin/tazobactam; Fos, fosfomycin; Nit, nitrofurantoin; Ert, ertapenem; Imi, imipenem; Ak, amikacin; Mem, meropenem.

**Table 4:** Comparation of antibiotic resistance and biofilm production in MDR and non-MDR isolates.

Variable	MDR (33)	Non-MDR (27)	*p* Value
Biofilm			
Biofilm production	33 (100%)	17 (62.5%)	<0.0001
Strong	15 (45.4%)	1 (5.8%)	<0.0001
Moderate	14 (42.4%)	7 (25.9%)	ns
Weak	4 (12.1%)	9 (33.3%)	0.0319
Negative	0 (0%)	10 (37.0%)	<0.0001

## Data Availability

Not applicable.

## Supplementary Materials

The following supporting information can be downloaded at https://www.mdpi.com/article/10.3390/pathogens12040537/s1, Table S1: Characteristics of 60 isolates antibiotic resistant.
