# Supplementary material for: Prevalence, Resistance Patterns and Biofilm Production Ability of Bacterial Uropathogens from Cases of Community-Acquired Urinary Tract Infections in South Italy
Source: Pathogens. 2023 Mar 29;12(4):537. doi: 10.3390/pathogens12040537 (PMC10145297; doi:10.3390/pathogens12040537)
Supplement: Supplementary file 1 [file pathogens-12-00537-s001.zip › pathogens-2271275-supplementary.pdf]

Table S1. Characteristics of 60 isolates antibiotic resistant

Isolates	Bacteria	Sex	Biofilm formation ability	Resistance profile	Antimicrobial resistance classification
-	*Escherichia coli* ATCC 25922	-	Strong		
-	*Klebsiella pneumoniae* ATCC 13883	-	Moderate		
-	*Proteus mirabilis* ATCC 35659	-	Moderate		
126E	*Escherichia coli*	Female	Weak	Amp; Nor;Amc; Caz; Gm; Ert	MDR
15E	*Escherichia coli*	Female	Moderate	Amp; Amc; Ctx; Caz; Gm; Mem	MDR
102E	*Escherichia coli*	Female	Moderate	Amp; Amc; Ctx; Caz; Gm;	MDR
75E	*Escherichia coli*	Male	Moderate	Amp; Amc; Ctx; Caz; Gm;	MDR
26E	*Escherichia coli*	Male	Strong	Amp; Nor; Amc; Cip; Ctx; Caz; Gm; Ert	MDR
12E	*Escherichia coli*	Female	Moderate	Amp; Amc; Cip; P/T; Gm	MDR
184E	*Escherichia coli*	Female	Strong	Amp; Amc, Fos Ert	MDR
207E	*Escherichia coli*	Male	Negative	Amp; Amc; P/T	Non- MDR
151E	*Escherichia coli*	Female	Moderate	Amp; Amc; Sxt; Fos	MDR
286E	*Escherichia coli*	Male	Weak	Amp; Amc; Ctx; Caz; Ert	MDR
301E	*Escherichia coli*	Male	Moderate	Amp; Amc; Ctx; Caz;	Non- MDR
302E	*Escherichia coli*	Female	Negative	Amp; Amc; Gm	Non- MDR
325E	*Escherichia coli*	Female	Moderate	Amp; Amc; Cip	Non- MDR
401E	*Escherichia coli*	Female	Strong	Amp; Amc; Ctx; Caz; Gm; Ak	MDR
510E	*Escherichia coli*	Female	Strong	Amp; Amc; Ctx; Caz; Gm;	MDR
615E	*Escherichia coli*	Male	Strong	Cip; Ctx; Caz; Gm	Non- MDR
722E	*Escherichia coli*	Male	Negative	Nor; Cip; Gm	Non- MDR
723E	*Escherichia coli*	Male	Strong	Nor; Sxt; Cip; Gm; Ert	MDR
733E	*Escherichia coli*	Male	Weak	Amc; Cip; Gm; P/T	MDR
761E	*Escherichia coli*	Female	Moderate	Amp; Amc; Cip; Caz; Gm	MDR
66K	*Klebsiella pneumonise*	Female	Strong	Amp; Nor; Amc; Cip; Ctx; Caz; Gm; Ak	MDR
11K	*Klebsiella pneumonise*	Male	Strong	Amp; Nor; Amc; Stx; Cip; Ctx; Caz	MDR

13K	*Klebsiella pneumonise*	Female	Moderate	Amp; Amc; Cip; Ctx; Caz	MDR
27K	*Klebsiella pneumonise*	Male	Moderate	Amp; Cip; Stx; Ctx; Caz	MDR
33K	*Klebsiella pneumonise*	Male	Strong	Amp; Cip; Stx; Gm; Ak	MDR
105K	*Klebsiella pneumonise*	Female	Weak	Amp; Cip; Ctx; Caz	Non- MDR
60K	*Klebsiella pneumonise*	Female	Negative	Amp; Cip; Ctx; Caz	Non- MDR
41K	*Klebsiella pneumonise*	Female	Moderate	Amp; Cip; Ctx; Caz	Non- MDR
88K	*Klebsiella pneumonise*	Male	Moderate	Amp; Ctx; Caz	Non- MDR
15K	*Klebsiella pneumonise*	Female	Moderate	Amp; Cip; Ctx; Fos	MDR
47K	*Klebsiella pneumonise*	Male	Weak	Amp; Cip; Fos; Gm	MDR
51K	*Klebsiella pneumonise*	Male	Weak	Amp; Ctx	Non- MDR
83K	*Klebsiella pneumonise*	Male	Strong	Amp; Nor; Sxt; Caz; Gm	MDR
108K	*Klebsiella pneumonise*	Female	Moderate	Amp; Nor; Sxt	Non- MDR
93K	*Klebsiella pneumonise*	Female	Moderate	Amp; Sxt	Non- MDR
101K	*Klebsiella pneumonise*	Female	Negative	Amp; Sxt	Non- MDR
118K	*Klebsiella pneumonise*	Female	Strong	Amp: Cip; Ctx; Gm	MDR
12K	*Klebsiella pneumonise*	Female	Weak	Amp; Nor	Non- MDR
2K	*Klebsiella pneumonise*	Male	Moderate	Amp; Nor; Gm Fos; Ert	MDR
128K	*Klebsiella pneumonise*	Female	Weak	Amp	Non- MDR
11P	*Proteus mirabilis*	Male	Moderate	Amp; Amc;	Non- MDR
81P	*Proteus mirabilis*	Female	Strong	Amp; Amc; Cip; Ak; Mem	MDR
26P	*Proteus mirabilis*	Female	Moderate	Amp; Nit; Stx; Ctz; Caz Ert	MDR
71P	*Proteus mirabilis*	Male	Moderate	Amp; Amc; Stx; Ctx; Caz; Fos	MDR
28P	*Proteus mirabilis*	Female	Moderate	Amp; Amc; Stx; Gm	MDR
15P	*Proteus mirabilis*	Male	Strong	Amp; Amc; Stx; Ctx; Caz; Fos; Imi	MDR
33P	*Proteus mirabilis*	Female	Weak	Amc; Cip	Non- MDR
50P	*Proteus mirabilis*	Male	Weak	Cip; Stx	Non- MDR
42P	*Proteus mirabilis*	Female	Strong	Cip; Stx; Fos; Imi	MDR
37P	*Proteus mirabilis*	Female	Strong	Amc; Cip; Stx; Ak	MDR
90P	*Proteus mirabilis*	Female	Negative	Stx; Ctx; Caz	Non- MDR
63P	*Proteus mirabilis*	Female	Moderate	Stx; Ctx; Caz; Gm; Fos	MDR
25P	*Proteus mirabilis*	Female	Strong	Gm; Stx; P/T; Fos	MDR
6P	*Proteus mirabilis*	Male	Negative	Gm; Ak	Non- MDR
7P	*Proteus mirabilis*	Male	Negative	Amc; Cip	Non- MDR

62P	*Proteus mirabilis*	Female	Weak	Amp; Amc; Ak	Non- MDR
31P	*Proteus mirabilis*	Female	Negative	Amp; Amc	Non- MDR
55P	*Proteus mirabilis*	Male	Weak	Amc; Cip	Non- MDR
93P	*Proteus mirabilis*	Male	Weak	Amc; P/T; Fos	Non- MDR
1P	*Proteus mirabilis*	Female	Negative	Ert; Mem	Non- MDR

Abbreviation: MDR= Multidrug-resistant bacteria, Non-MDR= Non Multidrug-resistant bacteria; Amp, ampicillin; Nor, norfloxacin; Amc, amoxicillin/clavulanic acid; Cip, ciprofloxacin; Sxt, trimethoprim/sulfamethoxazole; Ctx, cefotaxime; Caz, ceftazidime; Gm, Gentamycin; Fep, Cefepime; P/T, Piperacillin/tazobactam; Fos, fosfomycin; Nit, nitrofurantoin; Ert, ertapenem; Imi, imipenem; Ak, amikacin; Mem, meropenem.
